# Knowledge, attitudes, and perceptions of pharmacogenetic testing in swiss community pharmacies: a mixed-methods study among physicians, pharmacists, and the general public

**DOI:** 10.3389/fphar.2026.1827201

**Published:** 2026-07-03

**Authors:** Aude Coumau, Tanguy Jung, Chantal Csajka, Julie Dubois

**Affiliations:** 1 Centre for Research and Innovation in Clinical Pharmaceutical Sciences, Lausanne University Hospital and University of Lausanne, Lausanne, Switzerland; 2 Institute of Pharmaceutical Sciences of Western Switzerland, University of Geneva, University of Lausanne, Geneva, Switzerland; 3 School of Pharmaceutical Sciences, University of Geneva, University of Lausanne, Geneva, Switzerland; 4 Institute of Family Medicine, University of Fribourg, Fribourg, Switzerland

**Keywords:** attitude, genetic testing, perception, pharmacies, pharmacist, pharmacogenetics, physician, precision medicine

## Abstract

Pharmacogenetic testing represents a promising approach for the individualization of drug therapies. Nevertheless, its integration within the Swiss healthcare system remains limited to date. This study aimed to explore the knowledge, attitudes, and perceptions of physicians, pharmacists, and the general public toward PGx testing conducted in community pharmacy settings in Switzerland. A mixed-methods design was employed. Structured questionnaires were used to assess participants’ knowledge, perceived clinical utility, and anticipated barriers to implementation, while semi-structured interviews allowed for a more in-depth exploration of individual perspectives. Quantitative data were analyzed using descriptive statistics, consistent with the exploratory scope of the study, and qualitative data were processed through thematic analysis. A total of 332 participants completed the questionnaire, including 100 physicians, 122 pharmacists, and 110 members of the general public, and 26 took part in the interviews. PGx testing was perceived as clinically relevant across all stakeholder groups. The main barriers identified included insufficient specialized training, the absence of standardized guidelines, financial constraints, and ethical concerns. The need for strengthened interprofessional collaboration was also highlighted. This study sheds light on the key challenges associated with the integration of PGx testing in community pharmacy practice in Switzerland, spanning educational, organizational, and ethical dimensions. The findings should be interpreted within a descriptive framework, as the study design does not support causal or inferential conclusions. To enable successful implementation, it appears essential to develop targeted training programs, structure interprofessional collaboration, and establish robust policies for the protection of genetic data.

## Introduction

Drug response variability is influenced by clinical, demographic, environmental, and genetic factors, with genetics alone accounting for 15%–30% of individual differences in drug response (Zhou et al., 2017; Weinshilboum and Wang, 2017). Pharmacogenetics (PGx) plays a key role in personalizing treatment to enhance drug efficacy and safety (Cecchin and Stocco, 2020). PGx tests can be done preemptively to reduce the risk of toxicity or ineffectiveness, or reactively to explain therapeutic failure or adverse drug events (Relling and Evans, 2015; Phillips et al., 2001). The Food and Drug Administration (FDA) now includes PGx information on over 200 drug labels. These recommendations support treatment selection (pharmacodynamic relevance of genetic variants influencing drug targets) and guide dose adjustments (pharmacokinetic relevance of variants affecting drug metabolism or transport), enabling clinicians to personalize both the choice and the dosing of therapies (U.S. Food and Drug Administration, 2022; Vivot et al., 2015; Chang et al., 2015).

Recent years have seen an intensification of international efforts to promote personalized medicine through policies aimed at standardizing medical practices and encouraging digital innovation (Collins and Varmus, 2015; ICPerMed, 2019a; PerMed EP., 2023; ICPerMed, 2019b; PerMed E., 2023). For example, within the European Union, 28 policies and an additional 23 at the member-state level (EU-MS) focus on personalized care, illustrating coordinated initiatives in this area (Beccia et al., 2022). PGx testing have already been implemented and evaluated in community pharmacies in countries such as the United States of America (Berenbrok et al., 2019), Canada (Papastergiou et al., 2017), and the Netherlands (van der Wouden et al., 2019). For example, a study conducted in Dutch community pharmacies revealed that 24.2% of newly prescribed medications were associated with actionable drug-gene interactions, leading to adjustments in pharmacotherapy and enabling more personalized treatment approaches based on PGx (van der Wouden et al., 2019).

While PGx testing is advancing in many countries, its adoption in Switzerland remains cautious. In most Swiss medical and pharmacy settings, genetic factors that could influence medication response are not routinely considered, except for certain high-risk drugs where genetic mutations increase the risk of severe toxicity. PGx testing is recommended for seven specific medications (abacavir (HLA-B*57:01), carbamazepine (HLA-A*31:01, HLA-B*15:02), azathioprine *(TPMT),* 6-mercaptopurine *(TPMT),* 5-fluorouracil *(DPYD),* capecitabine *(DPYD),* and irinotecan (UGT1A1*28)) before starting treatment, as genetic testing is both clinically and economically justified (CHUV. CHUV - Département oncologie, 2018; SSPT SSdPeTC. Société Suisse de Pharmacologie et Toxicologie Clinique SSPT, 2019). A new ordinance of the Genetic Analysis Act (LAGH) was introduced in 2022, allowing pharmacists to conduct PGx testing under specific legal conditions. Such testing is limited to analyses directly related to a prescribed medication and requires prior contact with the prescribing clinician, who retains full responsibility for any therapeutic adjustments (LAGH, 2022). Yet, PGx testing for the therapeutic management of drugs in Switzerland is not part of routine care, likely limited by the combination of factors that have to be fully investigated. There is a need to better understand the barriers to its implementation to favor its adoption.

To date, several studies have assessed the perceptions and interest of patients, pharmacists, and physicians regarding the offer or use of PGx testing in community pharmacy settings. These studies have highlighted multiple needs, including targeted education and training, strengthening professionals’ confidence in the interpretation of PGx results, the development of clinical recommendations and algorithms, and the implementation of clinical decision support (CDS) systems to translate genotypes into therapeutic recommendations. Moreover, the need for structured interprofessional collaboration (pharmacist-physician) and effective communication channels has been consistently identified (Rahawi et al., 2020; Stegelmeier et al., 2020; Gibson et al., 2017; Kobayashi and Satoh, 2010; De Denus et al., 2013; Writer et al., 2021; Haga et al., 2016; Bright et al., 2021; McMahon and Tucci, 2011; Tuteja et al., 2013; Alexander et al., 2014; Muzoriana et al., 2017; Haga et al., 2012; Haga et al., 2019). In parallel, pilot studies and proof-of-concept investigations have evaluated the feasibility of offering PGx testing in community pharmacy settings across several countries (e.g., the United States, Canada, and the Netherlands). Overall, these studies report high patient acceptability and satisfaction, as well as pharmacists’ operational capacity to integrate PGx testing and counselling into routine workflow, provided that interpretation support tools and a clearly structured service model are in place (Coumau et al., 2025).

In Switzerland, an evaluation of perceptions and implementation strategies showed that, although pharmacists are legally authorized to initiate PGx testing, such tests remain rarely performed in community pharmacy practice (Wiss et al., 2024). Identified needs include clarification of care pathways, access to clinical decision support tools, and the development of a sustainable reimbursement model (Wiss et al., 2024). Nevertheless, despite these advances, PGx has not yet been implemented at scale as a routine service in Swiss community pharmacies, and population-level data suggest that the use of PGx testing in ambulatory care remains limited.

Therefore, it is necessary to identify the specific needs of key stakeholders involved in the use of PGx testing in community pharmacy within the Swiss context, to support a realistic, scalable, and equitable implementation of PGx testing in community pharmacy practice. To that endeavor, we conducted an exploratory analysis of the opinions and knowledge of PGx, as well as the barriers and facilitators to its use in community healthcare settings by engaging various stakeholders potentially involved in PGx use.

## Methods

### Study design

This research used a multiple-method approach (Morse, 2003). The quantitative component consisted of structured questionnaires to evaluate participants’ knowledge and attitudes toward PGx testing. The qualitative component involved semi-structured interviews to assess participants’ intentions to use, experiences, and perceptions of PGx testing.

### Study population

The study targeted three groups: Swiss residents from the general population, pharmacists, and physicians practicing in Switzerland (for healthcare professionals). Participants had to be fluent in French for participating to the interviews.

### Data collection

#### Quantitative data collection

We developed three questionnaires to assess different aspects of PGx. The development of these questionnaires was based on the existing literature on the subject (Rahawi et al., 2020; Stegelmeier et al., 2020; Blagec et al., 2022) and tailored to the specific needs of the study’s target groups. We also used the questionnaire methodology (Boynton and Greenhalgh, 2004; Bradburn et al., 2004; Groves et al., 2011). To ensure content relevance and clarity, the questionnaires underwent internal face-validity assessment by the research team, which included one clinical pharmacologist and pharmacists. Minor revisions were made to improve wording and alignment with the study objectives before distribution. No formal psychometric validation was conducted, as the aim of the study was exploratory. The elements of the questionnaires comprised the general awareness of PGx, its perceived clinical utility, familiarity with specific PGx applications, and potential barriers to its implementation. For the general population, the questionnaire included a concise introduction to PGx, explaining its role in personalizing medication and reducing adverse drug reactions. Recruitment was conducted through local medical and pharmacy societies in four cantons (Vaud, Valais, Genève, Fribourg), the Swiss Network for Primary Care Physicians (Réseau-delta.ch), and the Swiss Pharmacists Association (Pharmasuisse). Regarding Swiss residents, questionnaires were distributed via community pharmacies, the Swiss Patient Safety Foundation (securitedespatients.ch), and through social media promotion. A REDCap link to the questionnaires was sent by email to physicians, pharmacists, and to residents. This email provided an overview of the study, explaining its framework, objectives, and the significance of understanding perceptions of PGx testing. An information sheet was also provided, offering further details on the study’s background and goals to ensure informed participation. To maximize responses, three follow-up emails were sent at 1-month intervals. Data collection ran from February to May 2023.

#### Qualitative data collection

Participants who completed the surveys and consented to further participation were invited to take part in semi-structured interviews. The objective was to conduct 10 interviews per group (Swiss residents, physicians and pharmacists) and the first respondents from each category were selected. The final number of interviews was adjusted based on data saturation, ensuring that no new significant insights or themes emerged, thereby providing a comprehensive understanding of the topics explored.

The interviews were conducted between July and October 2023 b y videoconference or in face-to-face meetings. Interview guides were tailored to each stakeholder group, focusing on PGx opinions, barriers, and factors encouraging its adoption in pharmacy practice. For Swiss residents, a definition of PGx was provided at the beginning of the interview to ensure a common understanding. Face-validity was assessed through internal pilot testing of the guides with two members of the research team to ensure clarity, coherence, and adequacy of the questions. Minor refinements were made prior to data collection. The interview guides were structured and adapted from the Exploration phase domains of the Framework for the Implementation of Services in Pharmacy (FISpH), ensuring that key elements relevant to the early stages of service implementation were systematically explored across stakeholder groups (e.g., value assessment, organizational fit, community fit) (Moullin, 2016). Participants signed a consent form before each interview to allow for audio-recording, which was transcribed verbatim by one researcher and then reviewed for accuracy by another investigator. Interview guides are provided in Supplementary Materials (Tables 1–3).

### Data analyzes

Descriptive statistical analyses of participants’ characteristics, including age, sex, place of practice, and other relevant variables, were performed using Stata (StataIC 16). The questionnaires were analyzed using response proportions. Items consisted of either binary questions or multiple-response questions. A 7-point Likert scale was used for multiple-response items to allow respondents to express nuanced attitudes and to reduce central-tendency bias. To facilitate interpretation, the response categories collapsed as follows: strongly agree and agree were combined into agree; indifferent was kept as a standalone category; somewhat agree and somewhat disagree were grouped together; and disagree and strongly disagree were combined into disagree. For continuous-response questions, median values were calculated, accompanied by their corresponding minimum and maximum values.

The transcripts of the interviews were imported into MAXQDA (VERBI 22.1.1). They were thematically analyzed (Braun and Clarke, 2006). Transcripts were independently coded by three researchers and an expert in qualitative sciences. The researchers compared their coding and collaboratively developed a codebook, which was iteratively adapted as new codes emerged from the transcripts. The codebook was then used to label the content of all subsequent transcripts, while performing multiple cross-checks to ensure the validity and rigor of the coding. Subsequently, codes were grouped into sub-themes and then into main themes. The qualitative analysis followed a two-step approach combining inductive thematic exploration and deductive theoretical alignment. In a second step, these emergent themes were mapped deductively onto the Exploration phase domains of the Framework for the Implementation of Services in Pharmacy (FISpH) (Moullin, 2016). This mapping ensured theoretical coherence and enabled interpretation of the findings within a validated implementation framework. The Consolidated Criteria for Reporting Qualitative Research (COREQ) checklist was followed to present the study results (Tong et al., 2007). The labels D for doctors, P for pharmacists, and G for the general population were assigned.

### Data conservation and ethical considerations

The study did not require approval from the Cantonal Commission on Ethics in Human Research (CER-VD) and began after authorization from the medical direction of the University Hospital of Lausanne. Although CER-VD (Req-2022–00458) excluded the need for formal approval, all procedures followed the Swiss Federal Act on Research involving Human Beings and the Helsinki Declaration.

## Results

One hundred physicians, 122 pharmacists, and 110 Swiss residents participated in the survey study and a total of 31 physicians, 40 pharmacists, and 30 members of the Swiss general population volunteered for the interviews. Ultimately, 28 participants (9 pharmacists, 9 physicians, and 8 members of the general population) were interviewed. The average interview duration was 23 min for the general population and pharmacists, and 25 min for physicians. Most interviews were conducted via videoconferencing, with four held on-site and two at the participants’ workplace. The descriptive characteristics of participants from both the questionnaire and interview phases are reported in Table 1.

**TABLE 1:** Descriptive characteristics of included physicians, pharmacists, and Swiss residents.

Characteristics n (%)	Physicians		Pharmacists		Swiss residents	
	Surveys (n = 100)	Interviews* (n = 9)	Surveys (n = 122)	Interviews* (n = 9)	Surveys (n = 110)	Interviews* (n = 8)
Gender (female)	49 (59)	3 (33)	89 (74)	5 (56)	48 (44)	2 (20)
Age (year) med [min-max]	42 [23–79]	49 [39; 65] *(missing value =3)*	39 [23–67]	40 [35; 49] *(missing value =1)*	29 [19–73]	31 [24; 75] *(missing value =2)*
Specialty GP* Psychiatrist Community pharmacy Other	51 (53) 13 (14) NA 32 (33)	0 5 (56) NA 4 (44)	NA NA 119 (98) 0 (0)	NA NA 7 (78) 2 (22)	NA	
Place of practice French-speaking German-speaking Italian speaking	44 (98) 1 (2) 0	9 (100) 0 0	49 (61) 28 (35) 3 (4)	8 (89) 1 (11) 0	NA	
Years of practice med [min-max]	18 [0–50]	22 [11; 36] *(missing value =2)*	11 [0–41]	10 [7; 22] *(missing value =2)*	NA	
Position Physician assistant Senior physician Chief physician Private practice physician	27 (28) 12 (12) 14 (14) 55 (56)	0 0 5 (56) 4 (44)	NA			
Pharmaceutical specialty Community pharmacy Other	NA		119 (98) 0	7 (78) 2 (22)	NA	
Worked in clinical pharmacology department (yes)	11 (11)	1 (14) *(missing value =2)*	NA			
PGx* course at university (yes)	31 (32)	1 (14) *(missing value =2)*	50 (42)	4 (50) *(missing value =1)*	NA	
Having ever prescribed/use a PGx test (yes)	23 (24)	2 (29) *(missing value =2)*	7 (6)	0 *(missing value =1)*		
Use PGx in practice (yes)	6 (6)	1 (14) *(missing value =2)*	NA			
Post graduate degree* (yes)	72 (73)	7 (100) *(missing value =2)*	53 (44)	2 (33) *(missing value =3)*	NA	
Educational background Compulsory Post-compulsory Higher	NA				4 (4) 19 (18) 85 (79)	0 1 (13) 5 (63)
Healthcare system rating Excellent Very good Good Fair Poor					7 (6) 33 (30) 51 (47) 10 (9) 8 (7)	1 (17) 2 (33) 2 (33) 0 1 (17) *(missing value =2)*

Values represent total numbers and percentages (%). Percentages are calculated using the number with available data, missing data are specified in the table. For continuous variables, median (med), range [min-max]. Interviews* refers to semi-structured interviews; GP* stands for general practitioner. Post-graduate degree here refers to a professionally oriented advanced qualification obtained after completion of a university degree, typically involving structured continuing education and/or federally regulated postgraduate training (FMH: swiss medical association responsible for regulating postgraduate medical training and awarding federally recognized specialist titles; FPH: swiss professional body responsible for postgraduate and continuing education in community pharmacy, awarding advanced pharmacy qualifications; CAS: certificate of advanced studies; a university continuing-education programme worth ≥10 ECTS, credits; DAS: diploma of advanced studies; a university continuing-education qualification requiring ≥30 ECTS, credits); Pharmacy setting* includes neighborhood and city center pharmacies. The educational level includes compulsory, post-compulsory (vocational diploma, high school), and higher education (Bachelor’s/Master’s), PGx: Pharmacogenetics.

The analysis of the perspectives of physicians, pharmacists and the general population regarding PGx testing, derived from both questionnaire responses and semi-structured interviews, identified a total of 6 themes: knowledge of PGx principles, clinical utility of PGx testing, integration of PGx testing, role and collaboration between physicians and pharmacists, economic aspects of PGx testing, and data confidentiality and ethics. A summary of the results is provided in Table 2, and detailed information is presented in Tables 3–5.

**TABLE 2:** Overview of key similarities and differences across the physician, pharmacist, and Swiss resident surveys.

Themes	Physicians	Pharmacists	Swiss residents	Overall interpretation
Knowledge of pharmacogenetic principles	The basic definition of PGx was generally well identified, but substantial uncertainty remained regarding practical aspects, such as turnaround time, medications with strong PGx recommendations, and pharmacists’ authorization to perform PGx tests	The basic definition of PGx was understood, and participants demonstrated slightly more familiarity with certain practical elements (e.g., turnaround time, manufacturer information), though substantial gaps persisted overall	Awareness was considerably lower: Only a minority had ever heard of PGx, even fewer could explain what PGx testing is used for, and only a very small portion were aware that specific medications require PGx testing in Switzerland	Healthcare professionals showed stronger theoretical knowledge than swiss residents, but practical and regulatory understanding remained limited across all groups
Clinical utility of pharmacogenetic testing	Most considered that PGx could improve pharmacotherapy	Most considered that PGx could improve pharmacotherapy	More than half believed that PGx could improve medication management. The most valued expected benefits were reduced adverse effects, increased effectiveness, and reduced trial and error	All groups perceived PGx as potentially beneficial for treatment optimization
Integration of pharmacogenetic testing	Expressed caution toward PGx integration, citing uncertainty about evidence strength, applicability of recommendations, interpretative complexity, and the time required for routine implementation	Showed cautious support, somewhat more favorable toward implementation tools. Reported uncertainty about interpretation, practical integration, and limitations in recommending tests, interpreting results, explaining them to patients, and providing feedback to clinicians	Only a minority expressed concerns that PGx testing could reveal genetic predispositions to disease, while nearly half did not share this apprehension, and the remainder were neutral	Healthcare professionals expressed a reserved position toward PGx integration, marked by uncertainties regarding the strength of the evidence, the applicability of existing recommendations, the complexity of interpretation, and the practical feasibility of routine implementation. Concerns about the potential revelation of genetic disease predispositions were infrequent among swiss residents
Role and collaboration between physicians and pharmacists	Supportive of pharmacists’ involvement in PGx activities; favorable toward pharmacists providing feedback on test results and proposing treatment-related solutions	Generally favorable to a role in PGx service delivery and interested in expanding services to include PGx testing, but acknowledged insufficient current knowledge for several PGx-related tasks	Generally supportive of a shared model of care: most trusted both physicians and pharmacists for PGx advice and were willing to share their PGx profile with both; only a small minority rejected community pharmacies as a setting for PGx testing	The three groups showed openness to pharmacist involvement in PGx: Physicians expressed a favorable view and supported pharmacists’ participation in PGx-related activities; pharmacists were motivated to develop PGx services while acknowledging their current skill limitations; and swiss residents were largely supportive of a shared model of care, trusting both physicians and pharmacists for PGx-related advice
Economic aspects of pharmacogenetic testing	More than half perceived PGx tests as expensive, and most felt that insurance coverage should be limited to cases supported by strong evidence	Less likely than physicians to view PGx tests as expensive, but still largely supportive of reimbursement only when supported by strong evidence	Mainly expected PGx tests to be covered by basic insurance, while also identifying cost as a major disadvantage	Economic considerations were a major concern across all groups. While support for reimbursement was high, it was generally conditional on demonstrated clinical value
Data confidentiality and ethics	Concerned about potential misuse of PGx data by insurance companies, while generally supportive of including PGx results in medical records, especially in electronic health records and physician files	Expressed concerns about potential insurance misuse of PGx data, though less strongly than physicians, and were supportive of storing PGx results in professional health records	Expressed strong concerns about data confidentiality; many were worried about insurers accessing PGx data, and confidentiality was the most frequently cited disadvantage of genetic testing	Data protection and potential misuse by insurers emerged as major concerns in all groups, particularly among swiss residents

PGx, refers to pharmacogenetics.

**TABLE 3:** Thematic structure of semi-structured interviews results.

FISpH exploration outcomes	Themes	Sub-themes	Illustrative codes (examples)
Community fit assessment	Knowledge of pharmacogenetic principles	Limited understanding of PGx *(G)*	​
Value assessment/Community fit assessment	Clinical utility of pharmacogenetic testing	Treatment personalization *(D/P/G)*	• Adaptation of treatment choice *(D/P/G)* • Reducing polypharmacy *(D/P/G)* • Treatment optimization *(D/P/G)* • Limiting trial-and-error prescribing *(D/P/G)* • Dose adjustment *(D/P/G)*
		Enhanced efficacy and safety *(D/P/G)*	• Reducing patient suffering *(D/G)*
		Improved clinical understanding for practitioners *(D/P)*	• Improved interpretation of treatment response *(D/P)* • To explain adverse effects *(D/P)* • Improved understanding of treatment failure *(P)*
		Time savings in daily practice *(D/P/G)*	• Avoiding delays in identifying non-response *(D/P)* • Reduction of time lost in dose adjustment *(D)* • Avoiding prolonged ineffective treatment *(D)*
		Lifelong applicability of PGx testing *(D)*	• Stability of PGx results over a lifetime *(D)*
		Gap between PGx theory and clinical practice *(D/P)*	• Influence of non-genetic factors on treatment response *(D/P)*
Organisational capacity assessment/Community fit assessment	Integration of pharmacogenetic testing	Practical conditions for PGx integration	
		• Time and workflow integration *(D/P)*	• Need for simple and time-efficient PGx procedures *(D)* • Need for seamless integration into daily workflow *(D)* • Need to preserve time for patient listening *(P)* • Time constraints as a major barrier *(P/G)* • Need for rapid PGx turnaround time *(D)*
		• Communication with patients *(D/P)*	• Structured and transparent patient-professional communication *(D/P)* • Patient education to improve understanding and reduce fears *(D/P)* • Providing accessible communication tools and public information *(P)*
		• Interpretation of test results *(D/P/G)*	• Need for clinical decision-support tools for PGx interpretation *(D/P)*
		• Computerised access to PGx information *(D/P/G)*	• Unified and interoperable systems for PGx data *(D/P)* • Integration of PGx results into the EHR *(D/P/G)* • Storage responsibility, access control and governance *(D/P/G)*
		• Healthcare professional education requirements *(D/P)*	• Need for coordinated frameworks and guidelines *(D/P)* • Need for incentive-based continuing education *(D/P)* • Need for clinician awareness, examples, and practical applicability *(D/P)*
Value assessment/Community fit assessment	​	Perceived benefits	
		• economic and cost-related impacts of personalized care (D/P/G)	• cost reduction through optimization of treatment *(D/G)* • long-term economic benefits *(P)*
Organisational capacity assessment		Perceived barriers	• complexity in interpreting PGx results *(D/P)* • insufficient knowledge and training *(D/P)* • cost-related constraints (D/P) • insufficient collaboration between physicians and pharmacists (P)
Organisational fit assessment/Organisation capacity assessment/Community fit assessment	Role and collaboration between physicians and pharmacists	PGx test execution	• The pharmacist’s essential role in conducting the PGx test *(D/P)* • The need for a serious, confidential setting that enables dialogue when performing the test *(D/G)* • Reluctance to perform the test in a pharmacy setting *(D/P/G)* • Reluctance to perform the test in a medical practice *(D/P/G)* • Requirement for competence and professionalism when performing the test *(D/G)* • The logical and practical choice of conducting the test in a pharmacy, the patient’s natural point of contact *(D/P/G)*
		Interpretation of PGx test results	• Essential physician-pharmacist collaboration for the interpretation and clinical integration of pharmacogenetics *(D/P)* • Physician advantage: Access to the full medical record, allowing for an integrated interpretation of pharmacogenetic results *(P)* • Possibility of involving a genetics specialist for interpretation *(P/G)*
		Use of PGx to guide treatment selection and therapeutic adjustments	• Pharmacist’s essential role in identifying therapeutic problems and alerting the physician *(D)* • Pharmacist’s role in proposing therapeutic adjustments based on genetic information *(P)* • Pharmacist’s role as a centralizer of data and a support for therapeutic choices *(D)* • Physician’s decision-making role based on the pharmacist’s recommendations *(P)*
Organisational capacity assessment/Community fit assessment		Patient communication of PGx test results	• Explanations of the PGx test results from the professionals performing the genetic test *(G)* • Physician’s role in explaining the results *(P/G)*
Service assessment/Community fit assessment	Economic aspects of pharmacogenetic testing	Economic aspect of the PGx test	• a short-term non-profitable investment that yields long-term benefits through treatment optimization *(G)*
		Potential reimbursement for the PGx test	• coverage by basic insurance *(D/P/G)* • coverage by supplementary insurance *(G)* • reimbursement is justified if the PGx test demonstrates a proven benefit *(D)* • conditional reimbursement depending on the cost of the treatment *(G)* • patients should not have to pay for the tests *(D/P)*
		Alternative options for financing PGx tests	• patients can pay for the tests *(G)*
Service assessment/Community fit assessment	Data confidentiality and ethics	Concerns about the use of PGx data	• Concerns about the privacy and control of personal data *(G)* • Concerns about insurers using health data in a discriminatory way *(D/G)*
		Medico-legal aspects and legal framework	• Legal-ethical issues in obtaining informed consent from incapacitated patients *(D)*
		Data confidentiality and security	• Limiting access to health data to only those professionals directly involved in the patient’s care *(D/G)* • Recognition of the patient’s right to their data *(D/P)* • Agreement to include the data in the medical record under strict confidentiality and secure access conditions *(G)* • Genetic data perceived as highly confidential *(D)*

PGx, refers to pharmacogenetics. D = doctors, P = pharmacists, G = general population, HER: Electronic Health Record. FISpH refers to the Framework for the Implementation of Services in Pharmacy, a pharmacy-specific implementation framework operationalized by Joanna C. Moullin and colleagues (Moullin, 2016).

**TABLE 4:** Detailed responses from the questionnaires addressed to physicians and pharmacists.

FISpH exploration outcomes	Themes	Questions and possible answers *(Correct answer in italics)*	Physicians number (%) < N = 100	Pharmacists number (%) N = 122
Organisational fit assessment	Knowledge of pharmacogenetic principles	1. What is pharmacogenetics?^a=^ ^1b=0^		
		*a. Studies genetic factors*	*97 (98)*	*116 (95)*
		b. Identifies genetic predisposition	0	4 (3)
		c. I do not know	2 (2)	2 (2)
		2. The typical time between buccal sample collection by the practitioner and receiving pharmacogenetic test results is^a=^ ^1b=0^		
		a. 3 months	16 (16)	4 (3)
		*b. 1 week*	*31 (31)*	*49 (40)*
		c. A few minutes	2 (2)	18 (15)
		d. I do not know	50 (51)	51 (42)
		3. A pharmacogenetic description is provided by the manufacturer for certain drugs (as per swissmedic info or compendium)^a=^ ^1b=0^		
		*a. True*	*48 (49)*	*82 (67)*
		b. False	9 (9)	10 (8)
		c. I do not know	42 (42)	30 (25)
		4. A genetic variation can influence the pharmacokinetics and/or pharmacodynamics of a drug^a=^ ^2b=1^		
		*a. True*	*98 (100)*	*119 (98)*
		b. False	0	0
		c. I do not know	0	2 (2)
		5. In Switzerland, there are 7 medications for which pharmacogenetic testing is strongly recommended *(Please identify 3 of these medications)* ^a=^ ^1b=0^		
		a. Clopidogrel, escitalopram, and aripiprazole	6 (6)	13 (11)
		b. Ibuprofen, codeine, morphine	2 (2)	5 (4)
		*c. Abacavir, azathioprine, carbamazepine*	*32 (32)*	*38 (31)*
		d. I Knew a strong recommendation existed but did not know which three drugs	15 (15)	26 (21)
		e. I Did not know pharmacogenetic testing was required for these 7 drugs	44 (44)	40 (33)
		6. In Switzerland, pharmacists are authorized to conduct pharmacogenetic tests related to medical practice^a=^ ^1b=0^		
		*a. True*	*11 (11)*	*41 (34)*
		b. False	27 (27)	35 (29)
		c. I do not know	61 (62)	46 (38)
		7. A patient who is a poor metabolizer for a specific cytochrome is prescribed an active drug primarily metabolized by that cytochrome. What are the potential consequences?^a=^ ^1b=2^		
		a. Decreased adverse effect risk	10 (10)	9 (8)
		*b. Increased adverse effect risk*	*80 (81)*	*112 (93)*
		c. Decreased therapeutic effect	24 (24)	29 (24)
		*d. Increased therapeutic effect*	*63 (64)*	*86 (72)*
		e. None	1 (1)	1 (1)
		f. I do not know	2 (2)	0
Value assessment	Clinical utility of pharmacogenetic testing	8. Pharmacogenetics can improve pharmacotherapy^a=^ ^1b=0^		
		a. Agree	87 (88)	107 (88)
		b. Indifferent	0	2 (2)
		c. SWT agree/disagree	11 (11)	13 (11)
		d. Disagree	1 (1)	0
Value assessment	Integration of pharmacogenetic testing	9. I Believe it is useful to systematically review all prescriptions, integrating pharmacogenetic information^a=^ ^3b=1^		
		a. Agree	28 (29)	33 (27)
		b. Indifferent	9 (9)	8 (7)
		c. SWT agree/disagree	51 (53)	69 (57)
		d. Disagree	9 (9)	11 (9)
		10. The clinical utility of pharmacogenetics lacks sufficient evidence^a=^ ^5b=1^		
		a. Agree	2 (2)	0
		b. Indifferent	29 (31)	30 (25)
		c. SWT agree/disagree	31 (33)	46 (38)
		d. Disagree	33 (35)	(37)
		11. There are few applicable recommendations on drug selection and dosage related to genetic information^a=^ ^6b=0^		
		a. Agree	24 (26)	29 (24)
		b. Indifferent	22 (23)	31 (25)
		c. SWT agree/disagree	40 (43)	57 (47)
		d. Disagree	8 (9)	(4)
		12. There are few clinical experts in pharmacogenetics^a=^ ^5b=2^		
		a. Agree	34 (36)	39 (32)
		b. Indifferent	21 (22)	43 (36)
		c. SWT agree/disagree	35 (37)	36 (30)
		d. Disagree	5 (5)	2 (2)
Organisational capacity assessment		13. Pharmacogenetic training (basic genetic knowledge, legal frameworks, performing and interpreting tests) should be provided as*^a=^ ^2b=0^		
		a. Participation in conferences/seminars	62 (63)	74 (61)
		b. Continuing education courses online with credit validation	72 (74)	97 (80)
		c. Integrated into the university curriculum (Bachelor’s/Master’s)	83 (85)	98 (81)
		d. Other	3 (3)	1 (1)
		Specify^a=^ ^99b=121^		
		e. Postgraduate training	0	1 (100)
		f. Targeted oral communication or conference booth	1 (100)	0
		14. Pharmacogenetic tests are expensive^a=^ ^4b=0^		
		a. Agree	50 (52)	41 (34)
		b. Indifferent	24 (25)	39 (32)
		c. SWT agree/disagree	21 (22)	40 (33)
		d. Disagree	1 (1)	1 (2)
		15. Carrying out pharmacogenetic tests (sample collection and sending to the laboratory) would take time I do not have^b=0^		
		a. Agree	NA	27 (22)
		b. Indifferent		12 (10)
		c. SWT agree/disagree		60 (49)
		d. Disagree		23 (19)
		16. The turnaround time for pharmacogenetic test results is short^a=^ ^6b=5^		
		a. Agree	11 (12)	15 (13)
		b. Indifferent	48 (51)	60 (51)
		c. SWT agree/disagree	27 (29)	34 (29)
		d. Disagree	8 (9)	8 (7)
		17. Integrating pharmacogenetic test results into my current practice would take time I do not have^a=^ ^3b=1^		
		a. Agree	24 (25)	42 (35)
		b. Indifferent	12 (12)	25 (21)
		c. SWT agree/disagree	38 (39)	45 (37)
		d. Disagree	23 (24)	8 (7)
		18. Interpreting pharmacogenetic tests seems too complex^a=^ ^4b=0^		
		a. Agree	18 (19)	15 (12)
		b. Indifferent	14 (15)	14 (11)
		c. SWT agree/disagree	38 (40)	65 (53)
		d. Disagree	26 (27)	28 (23)
		19. An automated system integrating gene-drug interactions should be implemented to assist with prescribing^a=^ ^2b=1^		
		a. Agree	55 (56)	74 (61)
		b. Indifferent	6 (6)	10 (8)
		c. SWT agree/disagree	34 (35)	37 (30)
		d. Disagree	1 (3)	0
		20. There is insufficient training for physicians/pharmacists on pharmacogenetics^a=^ ^3b=0^		
		a. Agree	67 (69)	69 (57)
		b. Indifferent	5 (5)	13 (11)
		c. SWT agree/disagree	24 (25)	39 (32)
		d. Disagree	1 (1)	1 (1)
	Integration of pharmacogenetic testing	21. Are there any other factors that you believe hinder or, on the contrary, facilitate the use of pharmacogenetic tests in clinical practice?^a=^ ^92b=113^		
		Cost and reimbursement	2 (25)	2 (22)
		Time and workflow constraints	1 (12)	1 (11)
		Training and competencies	2 (25)	2 (22)
		Interprofessional collaboration	1 (12)	1 (11)
		Administrative and regulatory burden	0	3 (34)
		Accessibility and availability of tests	1 (12)	0
		Organisation of care/local implementation	1 (12)	0
Community fit assessment		22. I Am concerned that patients do not understand that pharmacogenetic tests do not reveal predispositions to other diseases^a=^ ^3b=2^		
		a. Agree	25 (26)	31 (26)
		b. Indifferent	10 (10)	18 (15)
		c. SWT agree/disagree	47 (48)	53 44)
		d. Disagree	15 (15)	18 (15)
		23. Patients need to be educated and made aware of precision medicine^a=^ ^6b=0^		
		a. Agree	38 (40)	65 (53)
		b. Indifferent	11 (12)	15 (12)
		c. SWT agree/disagree	43 (46)	40 (33)
		d. Disagree	2 (2)	2 (2)
Organisational fit assessment	Role and collaboration between physicians and pharmacists	24. Community pharmacies are not the right place to prescribe, process, interpret, and advise on pharmacogenetic tests^b=1^		
		a. Agree	NA	3 (2)
		b. Indifferent		10 (8)
		c. SWT agree/disagree		61 (50)
		d. Disagree		47 (39)
		25. My current knowledge in pharmacogenetics does not allow me to recommend pharmacogenetic testing when necessary^b=0^		
		a. Agree	NA	68 (56)
		b. Indifferent		7 (6)
		c. SWT agree/disagree		38 (31)
		d. Disagree		9 (7)
		26. Pharmacists could prescribe pharmacogenetic tests in community pharmacies^a=^ ^1^		
		a. Agree	32 (32)	NA
		b. Indifferent	20 (20)	
		c. SWT agree/disagree	35 (35)	
		d. Disagree	12 (12)	
Organisational fit assessment	Role and collaboration between physicians and pharmacists	27. Pharmacists could interpret pharmacogenetic test results^a=^ ^1^		
		a. Agree	36 (36)	NA
		b. Indifferent	18 (18)	
		c. SWT agree/disagree	34 (34)	
		d. Disagree	11 (11)	
		28. Pharmacists could discuss pharmacogenetic test results with patients^a=^ ^1^		
		a. Agree	36 (36)	NA
		b. Indifferent	10 (10)	
		c. SWT agree/disagree	42 (42)	
		d. Disagree	11 (11)	
		29. Pharmacists could educate patients about pharmacogenetic test results^a=^ ^1^		
		a. Agree	43 (43)	NA
		b. Indifferent	13 (13)	
		c. SWT agree/disagree	33 (33)	
		d. Disagree	10 (10)	
		30. Pharmacists could provide feedback on pharmacogenetic test results^a=^ ^1^		
		a. Agree	68 (69)	NA
		b. Indifferent	8 (8)	
		c. SWT agree/disagree	18 (18)	
		d. Disagree	5 (5)	
		31. Pharmacists could propose appropriate solutions based on patient’s pharmacogenetic test results^a=^ ^1^		
		a. Agree	68 (69)	NA
		b. Indifferent	11 (11)	
		c. SWT agree/disagree	17 (17)	
		d. Disagree	3 (3)	
		32. I Believe the healthcare professional responsible for communicating pharmacogenetic test results to patients should be*^a=^ ^2b=1^		
		The pharmacist	55 (56)	95 (79)
		The physician	83 (85)	100 (83)
		A specialized geneticist or genetic counsellor	53 (54)	71 (59)
		Other	3 (3)	2 (2)
		Specify^a=^ ^99b=120^		
		A trained professional (physician or pharmacist)	0	1 (50)
		The professional performing the test (physician, pharmacist, or geneticist)	0	1 (50)
		Advanced practice nurses (APNs)	1 (100)	0
Organisational capacity assessment		33. My current knowledge does not allow me to collect a saliva sample^b=0^		
		a. Agree	NA	30 (25)
		b. Indifferent		5 (4)
		c. SWT agree/disagree		41 (34)
		d. Disagree		46 (38)
		34. My current knowledge in pharmacogenetics does not allow me to understand and interpret genetic test results^b=3^		
		a. Agree	NA	33 (28)
		b. Indifferent		6 (5)
		c. SWT agree/disagree		58 (49)
		d. Disagree		22 (18)
		35. It is difficult to discuss genetic test results with pharmacists^a=^ ^1^		
		a. Agree	19 (19)	NA
		b. Indifferent	34 (34)	
		c. SWT agree/disagree	32 (32)	
		d. Disagree	14 (14)	
		36. My current knowledge of pharmacogenetics does not allow me to provide a report to the patient in order to explain the results of their pharmacogenetic test^b=1^		
		a. Agree	NA	37 (31)
		b. Indifferent		5 (4)
		c. SWT agree/disagree		54 (45)
		d. Disagree		25 (21)
		37. My current knowledge does not allow me to give feedback to physicians on drug-gene interactions^b=0^		
		a. Agree	NA	33 (27)
		b. Indifferent		9 (7)
		c. SWT agree/disagree		52 (43)
		d. Disagree		28 (23)
		38. I Am interested in expanding my services by offering pharmacogenetic testing in my pharmacy^b=0^		
		a. Agree	NA	81 (66)
		b. Indifferent		8 (7)
		c. SWT agree/disagree		28 (23)
		d. Disagree		5 (4)
Service assessment	Economic aspects of pharmacogenetic testing	39. Regarding the management of pharmacogenetic tests, I believe that: health insurance (basic or supplementary) should cover all pharmacogenetic tests^a=^ ^2b=2^		
		a. Agree	20 (20)	35 (29)
		b. Indifferent	15 (15)	12 (10)
		c. SWT agree/disagree	42 (43)	58 (48)
		d. Disagree	21 (21)	15 (12)
		40. Regarding the management of pharmacogenetic tests, I believe that: health insurance (basic or supplementary) should only cover pharmacogenetic tests with strong evidence^a=^ ^2b=0^		
		a. Agree	82 (84)	91 (75)
		b. Indifferent	3 (3)	6 (5)
		c. SWT agree/disagree	11 (11)	19 (16)
		d. Disagree	2 (2)	6 (5)
Service assessment	Data confidentiality and ethics	41. Regarding the confidentiality of pharmacogenetic data, I believe this data should be included in the following medical records*^a=^ ^1b=1^		
		a. Included in the patient’s electronic health record	76 (77)	107 (88)
		b. Included in the physician’s file	77 (78)	89 (74)
		c. Included in the pharmacy’s file	71 (72)	86 (71)
		d. Only in the patient’s possession	12 (12)	14 (12)
		e. Other	1 (1)	1 (1)
		Specify^a=^ ^100b =121^		
		• According to the patient’s choice	0	1 (100)
		42. I Am concerned that insurance companies may misuse pharmacogenetic data^a=^ ^2b=2^		
		a. Agree	56 (57)	51 (42)
		b. Indifferent	9 (9)	14 (12)
		c. SWT agree/disagree	26 (27)	38 (32)
		d. Disagree	7 (7)	17 (14)

a and b represent missing values for physicians and pharmacists, respectively. Some responses were collected using a 7-point Likert scale and grouped as follows: Agree = strongly agree + agree; Indifferent; SWT, agree/disagree = somewhat agree + somewhat disagree; Disagree = disagree + strongly disagree. *: Multiple-choice questions: respondents could select more than one option, which may result in total percentages exceeding 100%. “I do not know” responses are included for certain questions to reflect uncertainty or lack of knowledge among the respondents. Open-ended questions are indicated by “Specify” option where respondents provided additional comments or answers not listed in the provided choices. FISpH refers to the Framework for the Implementation of Services in Pharmacy, a pharmacy-specific implementation framework operationalized by Joanna C. Moullin and colleagues (Moullin, 2016).

**TABLE 5:** Detailed responses from the questionnaires addressed to Swiss residents.

FISpH exploration outcomes	Themes	Questions and possible answers	n (%) N = 111
Community fit assessment	Knowledge of pharmacogenetic principles	1. I Have already heard of pharmacogenetics^a=^ ^0^	
		Yes	45 (41)
		2. I Know what a pharmacogenetic test is used for^a=6^	
		Yes	29 (28)
		3. In Switzerland, there are 7 medications for which a pharmacogenetic test is recommended^a=4^	
		Yes	4 (4)
Community fit assessment	Clinical utility of pharmacogenetic	4. I Prefer other methods than genetics to optimize my treatment^a=2^	
		a. Agree	17 (16)
		b. Indifferent	22 (20)
		c. SWT agree/disagree	27 (25)
		d. Disagree	43 (39)
Value assessment		5. Pharmacogenetics improves my medication management^a=2^	
		a. Agree	59 (54)
		b. Indifferent	14 (13)
		c. SWT agree/disagree	28 (26)
		d. Disagree	8 (7)
		6. Advantages of pharmacogenetic tests* *(Please select maximum 4 elements that are the most important for you*)^a=4^	
		Reduced trial and error	75 (70)
		Increased effectiveness	76 (71)
		Reduced adverse effects	87 (81)
		Confidence in medications	42 (39)
		Improvement in my medication adherence	41 (38)
		Improved willingness to try a new medication	34 (32)
Community fit assessment	Integration of pharmacogenetic testing	7. Concerns about pharmacogenetic tests revealing genetic predispositions^a=2^	
		a. Agree	19 (17)
		b. Indifferent	15 (14)
		c. SWT agree/disagree	24 (22)
		d. Disagree	51 (47)
Organisational fit assessment	Role and collaboration between physicians and pharmacists	8. Pharmacogenetic tests are too specialized for community pharmacists^a=3^	
		a. Agree	11 (10)
		b. Indifferent	30 (28)
		c. SWT agree/disagree	31 (29)
		d. Disagree	36 (33)
		9. Community pharmacists cannot interpret pharmacogenetic tests^a=5^	
		a. Agree	17 (16)
		b. Indifferent	30 (28)
		c. SWT agree/disagree	31 (29)
		d. Disagree	28 (26)
		10. Community pharmacies are the right place for pharmacogenetic tests^a=4^	
		a. Agree	28 (26)
		b. Indifferent	41 (38)
		c. SWT agree/disagree	32 (30)
		d. Disagree	6 (6)
Community fit assessment		11. I Trust the advice of^a=2^	
		a. Physician	35 (32)
		b. Pharmacist	3 (3)
		c. Both	67 (61)
		d. Neither	4 (4)
Community fit assessment	Role and collaboration between physicians and pharmacists	12. I Would share my pharmacogenetic profile with^a=5^	
		a. Physician	23 (22)
		b. Pharmacist	3 (3)
		c. Both	71 (67)
		d. Neither	8 (8)
	Economic aspects of pharmacogenetic testing	17. If I had to pay for a pharmacogenetic test^a=3^	
		a. Paid by basic insurance	84 (78)
		b. Paid by supplementary insurance	19 (18)
		c. Pay myself	1 (4)
Community fit assessment	Data confidentiality and ethics	18. Concerns about insurance companies accessing data^a=2^	
		a. Agree	66 (61)
		b. Indifferent	6 (5)
		c. SWT agree/disagree	16 (15)
		d. Disagree	18 (19)
​	​	19. What do you consider to be the disadvantages of genetic testing?* *(Please select the 2 elements that are most important to you)* ^a=^ ^3^	
		a. Data confidentiality	72 (67)
		b. Complicated healthcare management	26 (24)
		c. Cost of pharmacogenetic tests	61 (57)
		d. Patient discrimination	38 (35)
		e. Other	7 (7)
		Specify^a=^ ^106^	
		• Time constraint	1 (20)
		• Increase in medical appointments	1 (20)
		• Anxiety related to disease discovery	1 (20)
		• Access to genetic coding information	1 (20)
		• Limits of scientific intervention	1 (20)

a represent missing values. Some responses were collected using a 7-point Likert scale and grouped as follows: Agree = strongly agree + agree; Indifferent; SWT, agree/disagree = somewhat agree + somewhat disagree; Disagree = disagree + strongly disagree. *: Multiple-choice questions: respondents could select more than one option, which may result in total percentages exceeding 100%. Open-ended questions are indicated by “Specify” option where respondents provided additional comments or answers not listed in the provided choices. FISpH refers to the Framework for the Implementation of Services in Pharmacy, a pharmacy-specific implementation framework operationalized by Joanna C. Moullin and colleagues (Moullin, 2016).

### Knowledge of pharmacogenetic principles

Physicians and pharmacists demonstrated a solid understanding of PGx principles. Most correctly identified that PGx examines genetic factors influencing drug response, and nearly all recognized that genetic variation affects both the pharmacokinetics and pharmacodynamics of medications. The vast majority also understood that a poor metabolizer prescribed an active drug primarily metabolized through the affected cytochrome pathway faces an increased risk of adverse effects and were aware of the potential for improved therapeutic efficacy in such cases. Knowledge gaps were, however, apparent in more specific areas. Fewer than half of physicians and approximately two-thirds of pharmacists were aware that manufacturers provide PGx information for certain drugs. Awareness of typical turnaround times for PGx results were even lower, with only a minority in each group knowing that results are generally available within a week. Around one-third of respondents correctly identified three of the seven medications for which PGx testing is strongly recommended in Switzerland, while a substantial proportion either lacked awareness of specific drugs or were unaware that such recommendations existed. Awareness of pharmacists’ authorization to perform PGx testing was very low among physicians and remained limited among pharmacists themselves. In the general population, familiarity with PGx was limited overall. While a notable minority had heard of PGx, fewer could explain the purpose of the test, and only a very small fraction knew that testing is required for certain medications in Switzerland.

These findings were corroborated by qualitative data: whereas healthcare professionals demonstrated a solid grasp of PGx concepts in the interviews, two participants from the general population were unable to provide a clear explanation of what PGx entails.

### Clinical utility of pharmacogenetic testing

All participant groups expressed strong confidence in the clinical utility of PGx, with nearly all physicians and pharmacists agreeing on its potential to enhance pharmacotherapy. Among the general population, the reduction of adverse drug events was most frequently cited as the primary benefit, followed by improved treatment effectiveness. These views were reinforced in the interviews, where participants emphasised PGx’s role in personalising treatment and optimising the benefit-risk balance.

*“Ideally, the goal would be to improve patient safety, minimize adverse effects and optimize the desired effect, the positive effect of the product. That's what it's all about - optimizing the benefits while minimizing the risks. I think that's clearly the main benefit for the patient.”* - D1

Physicians also valued PGx for reducing trial-and-error in drug selection.

*“It would avoid trying multiple medications and experiencing side effects or no effects at all, saving time.” –* D7

Both physicians and pharmacists noted its potential to improve understanding of individual variability in drug metabolism, supporting more targeted therapeutic decisions.

*“ We are dealing with systems like blood pressure, diabetes, cholesterol, cardiovascular issues, and so on. I think there is really potential to explore here, which could also improve understanding by subsequently targeting only the relevant pathways.”–* D6

Across all groups, PGx integration was seen as a potential time-saving measure in routine practice.

*“I think we can save time because when we have patients for whom we conduct (…), we only adjust medication dosages if it’s truly justified, if we consider it justified. (…) And if we know upfront that the patient is a rapid metabolizer, we can anticipate things ahead of time. I believe this could save us some time.”* – P6

Several suggestions for optimizing PGx use were also raised. Physicians proposed tailoring tests to specific treatments or conditions, and one advocated for systematic testing of polymedicated patients. A few physicians and pharmacists suggested that a single lifetime test, ideally performed at birth, would offer lasting clinical benefit. However, some physicians cautioned that PGx provides only a partial picture of patient care, noting a gap between theoretical utility and clinical reality.

*“Beyond the presence or absence of a gene, I believe there is a whole regulatory process at the level of gene expression (…). You know as well as, if not better than, I do that numerous factors influence the penetrance of a trait at various levels.”* - D6

### Integration of pharmacogenetic testing

Physicians and pharmacists held mixed views on several aspects of PGx integration. While most did not consider the evidence supporting PGx clinical utility to be insufficient, opinions were divided regarding its inclusion in routine medication reviews, the adequacy of turnaround times, and the applicability of PGx-guided recommendations to drug selection and dosing. Both groups acknowledged the complexity of interpreting PGx results, and a clear majority agreed that current PGx training for healthcare professionals is insufficient. Accordingly, strong support was expressed for integrating PGx teaching into university curricula, online training modules with accredited certification, and automated prescription-assistance systems. Pharmacists also raised practical concerns around the time required to collect and process PGx samples, and both groups perceived testing as costly. Both physicians and pharmacists expressed nuanced concerns that patients might misinterpret the scope of PGx testing, particularly by conflating it with broader genetic disease prediction, whereas most members of the general population reported no such concerns.

In the interviews, participants identified several conditions for successful PGx integration. A recurring theme was the need for clear, reassuring communication with patients about what PGx testing involves and how it is conducted.

*“ I think that from the patient's perspective (…) I do not know enough about the subject, but I believe that when we talk about genetics, it can be intimidating. Therefore, it would be helpful for me to have a thorough understanding of the topic to effectively discuss the pros, the cons, and the fears that people might have regarding this (…).”* – P8

Physicians and pharmacists broadly agreed that test results should be easy to interpret, ideally through dedicated applications or algorithms, and that PGx data should be computerized and integrated into electronic health records.

*“There should be a standardized pharmacy system, and we would want a standardized patient record so that everyone can access this valuable information, but that is on a national scale.”* – D3

The need for a simplified, intuitive implementation framework was also emphasized.

*“We really need to find a simple and intuitive way for each physician to adjust medication doses, and so on, based on this genomic profile.”* – D4

Short turnaround times and adequate time allocation were identified as further prerequisites by physicians.

*“So, the test needs to be conducted with results available quickly (…).”* – D1

*“Definitely [there would be a time issue], because our administrative tasks are constantly increasing, and every second we spend away from the patient is… let’s say, every second we spend on these kinds of processes and maneuvers is ultimately time taken away from the patient.”* – D4

Professional training was widely regarded as a critical enabler.

*“(…) I think we need to set up e-learning, for example, where people can easily connect. And then it should be almost a prerequisite that pharmacists and doctors are trained to implement these procedures.”* – D5

Several benefits of integrating PGx testing were mentioned, the most widely discussed by most of all interviewees being the potential to reduce healthcare costs.

*“It will likely help reduce the costs of inadequate treatments prescribed to patients or reduce the amounts of medication”* – D5.

Financial barriers, however, emerged as the most significant obstacle, encompassing both the cost burden on patients and the investment required by pharmacies.

*“Costs, the investment it could entail in a pharmacy [would be a barrier to the implementation or use of PGx in practice]. Regarding reimbursement, it’s true that if we are the ones doing it currently, I think it would be charged to the patient, and that could be a barrier.”* – P9

Additional challenges included limited PGx awareness in the general population and concerns about the complexity of interpreting test results.

*“A lack of awareness among the general population. I have the impression that people generally think (…) that genetic testing can predict everything”* – P1

*“The challenge, as you pointed out, lies in interpreting the PGx results and determining how to adjust dosages accordingly.”*– P2

### Role and collaboration between physicians and pharmacists

Physicians and pharmacists held varied views on the pharmacist’s role in PGx testing. While most physicians acknowledged that pharmacists are well positioned to provide insights and recommend appropriate solutions based on test outcomes, only one-third believed they should prescribe tests, interpret results, or discuss them directly with patients. Among pharmacists, the majority considered community pharmacies appropriate settings for the full spectrum of PGx-related tasks; however, many acknowledged insufficient knowledge to confidently recommend testing, and reported lacking confidence in sample collection, result interpretation, report preparation, and physician feedback. Despite these limitations, a clear majority expressed interest in expanding their services to include PGx testing. Across all groups, there was general agreement that communicating results to patients could fall to a physician, a pharmacist, or a specialized genetic counsellor. In the general population, a substantial proportion did not consider PGx testing too specialized for community pharmacists, and many expressed trust in both physicians and pharmacists for this role.

In the interviews, opinions diverged on which professional should administer the test. Some physicians and pharmacists favored general practitioners or pediatricians, while others considered pharmacists the most appropriate.

*“(…) Well, patients trust their family doctor. So, if their family doctor presents the project by saying, 'Well, if you go to the pharmacy and your pharmacist offers it to you, go ahead,' they’ll do it. And generally, they also have quite a bit of trust in their pharmacist.”* – D5

Several members of the general population highlighted the practical convenience of community pharmacies.

*“I think the simplest option is in a pharmacy because I believe access is quite quick. The availability also seems fairly convenient to me.”* – G5.

Some physicians, however, noted that pharmacies may not be suitable for patients in nursing homes or with mobility issues, and both physicians and members of the general population emphasized the importance of the test being administered by a professional with adequate PGx expertise. A minority suggested hospitals as an alternative setting.

Regarding result interpretation, most physicians and pharmacists agreed this should be a collaborative process, drawing on the physician’s knowledge of diagnosis and treatment history and the pharmacist’s expertise in drug interactions.

*“I think we will be led to collaborate more and more, and I think it is extremely beneficial. I would say it is more of a two-person interpretation [physician and pharmacist], there is a discussion. We have to do it, it is still complex.”* – D7

On the communication of results to patients, participants across groups agreed that this responsibility could be assumed by either a physician or a pharmacist, provided the individual had received adequate training in the field.

*“I think it should be someone who is trained, because apparently this is a new field. So, someone who has undergone some form of training in this area, whether it's a pharmacist, a doctor, or someone else.”* – G5

### Economic aspects of PGx testing

Views on reimbursement were broadly consistent across groups. While only a minority of physicians and pharmacists supported full reimbursement of all PGx tests through basic health insurance, a clear majority favored coverage limited to tests backed by strong scientific evidence. Most members of the general population similarly supported reimbursement through basic insurance. In the interviews, participants across groups reinforced this position, citing the favorable cost-benefit ratio of PGx testing as a key justification.

*“Ideally, we can imagine that it would be reimbursed, because everyone stands to gain from it. In fact, if we manage to optimize the treatment, well, fewer side effects, less risk of hospitalization, or whatever else because something does not work with the medication, yes, I think it should be reimbursed. I do not think it should be the patient’s responsibility to pay.”*– P8

### Data confidentiality and ethics

Physicians and pharmacists strongly supported the integration of PGx information into medical records, favoring its inclusion in patients’ digital files, physicians’ records, and pharmacy files. However, concerns about data privacy were prominent across all groups. Many physicians and pharmacists worried about potential misuse of PGx data by insurance companies, a concern widely echoed in the general population. More broadly, data confidentiality was frequently cited as one of the principal disadvantages of genetic testing.

These concerns were extensively explored in the interviews. Most participants agreed that genetic data should be accessible to physicians, pharmacists, and patients.

*“So who [should have access to the test data and results?]: everyone involved in the medical chain, meaning the doctor, the pharmacist, and of course the patient, as it's obviously their right and property in my opinion.”* – P5

Conversely, there was broad consensus that insurance companies should be excluded from accessing genetic data, given concerns about discrimination based on genetic profiles.

*“There are negative aspects, and they must be taken into consideration from the start to prevent the negative side from taking over, such as the misuse of this kind of information in profiling, or things like that which could create segments of populations with this type of profile, etc., etc. So, I would say it is quite delicate to handle. (…) However, as with all good intentions, there is a negative side that should not be overlooked; it must be considered and precautions taken to prevent any negative outcomes in the future.”* – G5

Most general population participants stressed that PGx data should be properly protected against commercialization and misuse. In this context, several physicians and pharmacists highlighted the need for a clear legal framework governing data use and requiring written informed consent from all relevant stakeholders.

*“I think the main challenge is still the privatization of data, or rather the use of data afterwards. Then, as a Swiss person, I would say that, in general, I would trust the use of it, but it’s more, let’s say, what could (…) the potentially negative aspect I could see would be, for example, the sale to insurance companies, the commercialization of the data.”* – G10

## Discussion

To our knowledge, this is the first Swiss study to assess the perceptions of the general population, pharmacists, and physicians about the benefits, challenges, and clinical integration of PGx testing within the Swiss French-speaking healthcare system. The findings highlight key elements related to knowledge, perceptions, and barriers to PGx adoption in community pharmacies, and suggest that integration could be facilitated through strengthened training, simplified interpretation tools, and enhanced interprofessional collaboration. The main barriers identified include limited practical knowledge, the absence of clear clinical guidelines, economic considerations, and concerns related to genetic data confidentiality. To our knowledge, no previous study in the field of PGx has applied the FISpH framework, which provides significant methodological added value to our analysis.

Our results are broadly consistent with observations reported across several European countries. The most frequently identified barriers, insufficient training, lack of reimbursement, and fragmentation of information systems, mirror those documented in primary care and community pharmacy settings elsewhere (Coumau et al., 2025; Kiani et al., 2026; Thornley et al., 2021). The U-PGx project, a large-scale implementation study across seven countries, further demonstrated the technical feasibility of clinical decision support (CDS) systems integrated into routine clinical workflows, underscoring their potential to support PGx implementation in multinational and multilingual contexts (Blagec et al., 2018; Blagec et al., 2022).

Physicians and pharmacists demonstrated solid theoretical knowledge of PGx but were less comfortable with practical aspects such as turnaround times, manufacturer-provided PGx information, and national recommendations. This pattern is consistent with prior studies showing adequate understanding of general PGx concepts alongside persistent difficulties in translating this knowledge into concrete clinical scenarios (Rahawi et al., 2020; Muzoriana et al., 2017). For instance, Muzoriana et al. reported that although 97% of pharmacists and pharmacy students recognized that genetic variability influences drug response, only 26% correctly answered an applied question on warfarin dosing (Muzoriana et al., 2017). Among Swiss residents, awareness of PGx was limited, with many unable to explain the purpose of testing or identify medications requiring PGx in Switzerland, findings largely consistent with patient-focused literature (Stegelmeier et al., 2020; Gibson et al., 2017; Haga et al., 2016), and particularly with Stegelmeier et al., who reported that only 28.9% of pharmacy consumers felt they had a good understanding of PGx (Stegelmeier et al., 2020).

All stakeholder groups expressed a strongly positive perception of PGx’s clinical utility, viewing it as a tool enabling therapeutic personalization and reducing trial-and-error prescribing. This enthusiasm is well supported in the literature (Rahawi et al., 2020; Stegelmeier et al., 2020; De Denus et al., 2013; Writer et al., 2021; Haga et al., 2016; Bright et al., 2021; McMahon and Tucci, 2011; Tuteja et al., 2013; Haga et al., 2012; Haga et al., 2019). Rahawi et al. reported that over 80% of surveyed pediatricians believed PGx could enhance both efficacy and safety (Rahawi et al., 2020), while Tuteja et al. found that 87% of pharmacists perceived it as reducing adverse events and optimizing dosing (Tuteja et al., 2013). Patient-centred studies similarly show that interest in PGx is primarily driven by the desire to select the most effective treatment (Haga et al., 2016; Bright et al., 2021). Importantly, however, our qualitative findings reveal meaningful nuances: several physicians emphasized that PGx alone does not fully account for interindividual variability and should be applied selectively, for specific medications, conditions, or patient profiles, ideally within a pre-emptive testing framework. This view is echoed by Writer et al., who found that pharmacists advocated targeting PGx toward patients with the greatest clinical need, such as those who are polymedicated or experiencing therapeutic failure (Writer et al., 2021).

Regarding interprofessional roles, physicians generally held a positive perception of pharmacists’ involvement in PGx, recognizing their competence in ordering tests, interpreting results, and providing patient education, an aspect not previously described in the literature. At the same time, physicians acknowledged potential difficulties in discussing PGx results with pharmacists, a barrier documented by Wiss et al., who identified insufficient pharmacist-physician coordination and unstructured communication as key implementation obstacles (Wiss et al., 2024). Among pharmacists, perceived ability to recommend tests, interpret reports, and communicate with physicians remained low, consistent with studies highlighting limited confidence in PGx counselling and a strong need for additional training (De Denus et al., 2013; Writer et al., 2021; McMahon and Tucci, 2011; Tuteja et al., 2013). In this context, Stäuble et al. proposed a structured pharmacist-led PGx service framework, encompassing referral, pre-test counselling, testing, medication review, and follow-up, as an operational model to standardize practice (Stäuble et al., 2022). On the question of who should communicate results to patients, both physicians and pharmacists in our study felt this could appropriately involve physicians, pharmacists, or genetic specialists. O'Connor’s pilot study suggests that pharmacists should take the lead in communicating results and interpreting drug-gene interactions, while therapeutic adjustments remain the prescriber’s responsibility (O ‘Connor et al., 2012). Public perspectives were broadly consistent with this view, with participants expressing confidence in pharmacists’ interpretive abilities and trust in guidance from both professional groups (Haga et al., 2016; Haga et al., 2012).

Healthcare professionals widely supported the integration of PGx data into electronic health records (EHR), alongside the development of accessible, evidence-based CDS tools, needs well documented in the literature (Berenbrok et al., 2019; Writer et al., 2021; Wiss et al., 2024). As highlighted by Wiss et al., PGx integration into routine practice remains difficult without direct connectivity to clinical information systems and interoperable CDS tools (Wiss et al., 2024). Most physicians and pharmacists also considered their PGx training insufficient and supported its integration into both university curricula and e-learning formats, consistent with a broad body of literature emphasizing the need for applied, case-based training (Berenbrok et al., 2019; Rahawi et al., 2020; De Denus et al., 2013; McMahon and Tucci, 2011; Alexander et al., 2014; Muzoriana et al., 2017; Haga et al., 2019; Wiss et al., 2024; Moaddeb et al., 2015). On the question of time constraints, pharmacists in our study expressed relatively limited concern, a finding partially supported by Moaddeb et al., who found that communicating PGx results required less time in practice than anticipated (Moaddeb et al., 2015), though time pressure remains a widely reported barrier elsewhere (Writer et al., 2021; Alexander et al., 2014).

Reimbursement models emerged as a key determinant of PGx feasibility. Healthcare professionals broadly supported reimbursement but favored a selective approach tied to strong clinical evidence rather than universal coverage. Three broad strategies can be distinguished: pre-emptive reimbursement for medications with clearly established clinical utility; conditional pre-emptive testing where evidence is less robust, requiring comparison with alternative markers; and reactive reimbursement triggered by therapeutic failure or adverse drug reactions. While the first and third strategies are widely regarded as justified, the second remains more debated. Among the general population, our data indicates a preference for coverage through basic health insurance. Available health-economic evidence supports the hypothesis of medium-term financial benefit: Bain et al. reported substantial cost avoidance associated with PGx implementation, reaching nearly USD 200,000 under certain scenario assumptions (Bain et al., 2020). Gibson et al. further demonstrated that willingness to use PGx services is strongly dependent on insurance coverage, and that variability in willingness-to-pay risks introducing inequities if out-of-pocket financing prevails (Gibson et al., 2017). These findings align with the literature consistently identifying financial barriers as among the most significant obstacles to PGx integration (Writer et al., 2021; Alexander et al., 2014; O ‘Connor et al., 2012; Ferreri et al., 2014). Notably, several studies emphasize that the economic challenge extends beyond the cost of testing itself: O'Connor et al. stress the necessity of reimbursing the full service, interpretation, counselling, and coordination, to ensure sustainability (O ‘Connor et al., 2012), while Ferreri et al. illustrate that administrative barriers can block reimbursement even when patients accept billing (Ferreri et al., 2014).

Concerns related to data confidentiality and ethics emerged as central determinants of PGx acceptability. While physicians, pharmacists, and the general population largely supported sharing PGx information within the healthcare continuum, a clear boundary appeared regarding access by insurance providers, perceived as a major risk for discrimination and misuse. These concerns align with the Swiss regulatory framework, which classifies health and genetic information as sensitive personal data under the revised Federal Act on Data Protection, entailing rigorous protection requirements and restricted access (Federal Act on Data Protection FADP, 2020). This pattern is consistent with the broader literature, where concerns about secondary data use and access control represent primary barriers to genetic testing adoption (Kobayashi and Satoh, 2010; Bright et al., 2021; Tuteja et al., 2013; Muzoriana et al., 2017; Haga et al., 2012). Bright et al. further demonstrate that perceived risks associated with PGx are dominated by confidentiality concerns and fears about future consequences, such as insurance or employment implications (Bright et al., 2021). Our findings also reveal persistent confusion, across all groups, about the scope of PGx testing, particularly the belief that results might reveal predispositions to other diseases, underscoring the need for targeted counselling to prevent misunderstandings (Haga et al., 2012).

Our findings point to several actionable directions. Clinically, interprofessional coordination around PGx results appears essential to strengthen interpretation quality and promote harmonized therapeutic decisions. Integrating PGx data into interoperable EHR with embedded CDS tools would further support clinical decision-making and enhance patient safety. Economically, sustainable reimbursement models covering not only testing costs but also professional time for counselling, interpretation, and coordination are needed. Operationally, simplified and standardized testing pathways, including clear sample-collection procedures, reliable analysis platforms, and rapid result transmission into HER, would facilitate integration into community pharmacy workflows. Regarding data governance, transparent regulatory frameworks clearly defining access rights, informed consent procedures, and data-sharing limits, particularly with insurers, are essential. Finally, expanding PGx education at both undergraduate and postgraduate levels, supported by practice-oriented resources, remains a prerequisite for building healthcare professionals’ confidence and enabling sustainable PGx implementation.

Our study has several limitations that should be acknowledged. Voluntary participation may introduce a selection bias, as respondents may be more interested in PGx than their peers on average. Additionally, although demographic characteristics such as age, gender, and professional or educational background were collected, socioeconomic information was not assessed, limiting our ability to explore potential associations between socioeconomic factors and attitudes toward PGx testing. The combined use of questionnaires and semi-structured interviews allows for data triangulation, but social desirability and self-reporting biases remain possible, as participants may overestimate their knowledge or interest in PGx. Additionally, participants’ prior knowledge of PGx was not systematically assessed before the study, which may have influenced their responses and the interpretation of the results. The complexity of the topic may also have led to misunderstandings, particularly among the general population; however, since an explanation of the fundamental principles of PGx was provided at the beginning of the questionnaires and interviews, this risk has been reduced. Regarding the questionnaire development process, although the instruments were grounded in existing literature and subjected to internal face validity assessment by experts in clinical pharmacology and pharmacy practice, no formal psychometric validation was performed. This is consistent with the exploratory scope of the study, which sought to provide a preliminary overview of stakeholders’ knowledge, attitudes, and perceptions, rather than to produce a psychometrically validated measurement tool. Nonetheless, the lack of formal validation may compromise the reliability and interpretability of the quantitative results. Relatedly, the quantitative analysis relied primarily on descriptive statistics, which, while appropriate for an exploratory study, does not allow for the identification of potential statistical associations or the examination of factors that may explain the observed attitudes and perceptions. The findings should therefore be interpreted descriptively, as the study design does not support causal or inferential conclusions. Future studies should employ rigorous validation procedures, encompassing content validity, construct validity, and reliability testing, and incorporate inferential statistical analyses, such as regression models or association tests, to deepen the understanding of relationships between variables and strengthen the robustness and generalizability of the findings. Another limitation is that this study did not examine the technical modalities of PGx analysis, such as the choice between targeted gene panels and broader sequencing approaches (e.g., exome or whole-genome sequencing). While this was beyond the scope of our exploratory focus on perceptions and implementation barriers, the analytical strategy could have important consequences for cost, feasibility, interpretation, and long-term integration of PGx services. Finally, although the questionnaire was distributed across all regions of Switzerland, the sample was more representative of French-speaking Switzerland, which may introduce a regional bias. Nevertheless, the observed results appear consistent with those emerging from German-speaking Swiss contexts, where analogous implementation barriers have been reported, including gaps in professional education, insufficient integration of e-health technologies, and the lack of viable reimbursement structures (Wiss et al., 2024). This convergence across linguistic regions tentatively suggests that the obstacles identified may reflect broader, system-level challenges within the Swiss healthcare system. Furthermore, the diversity of respondents in terms of age, sex, and professional specialty covers a wide range of profiles, and this heterogeneity helps mitigate the potential effects of geographic bias, providing a relatively balanced picture of perceptions regarding PGx.

This study provides valuable insights into the implementation of PGx testing in community pharmacies in Switzerland. The results could significantly influence healthcare practices and authorities by providing data on the acceptance criteria and barriers to the implementation of PGx testing in community pharmacies, ultimately improving the personalization of treatments and patient care.

## Conclusion

While the study demonstrates a strong understanding of PGx among healthcare professionals and a positive outlook on its benefit, significant barriers remain. These include knowledge gaps in the general population, concerns about costs and data privacy, and a need for better training and infrastructure. Addressing these issues is crucial for the broader adoption of PGx in clinical practice. Further research should explore interventions to improve public education, enhance collaboration between physicians and pharmacists, and develop tools to support the integration of PGx into everyday healthcare.

## Data Availability

The raw data supporting the conclusions of this article will be made available by the authors, without undue reservation.
